# Supplementary material for: Advances in the Integrated Pest Management of Quinoa (Chenopodium quinoa Willd.): A Global Perspective
Source: Insects. 2024 Jul 18;15(7):540. doi: 10.3390/insects15070540 (PMC11276635; doi:10.3390/insects15070540)
Supplement: Supplementary file 1 [file insects-15-00540-s001.zip › insects-3081614-supplementary.pdf]

Table S1. Phytophagous arthropods that have been reported in quinoa fields in the world.

Order	Family	*Species*	Country
Orthoptera	Romaleidae	*Chromacris speciosa* (Thunberg)	Argentina
	Acrididae	*Dichroplus maculipennis* Blanchard	Argentina
		Rhammatocerus pictus (Bruner)	Argentina
	Gryllidae	*Gryllus assimilis* (Fabricius)	Bolivia
Hemiptera	Aleyrodidae	*Bemisia tabaci* (Gennadius)	Egypt
	Cicadellidae	*Anacuerna centrolinea* (Melichar)	Bolivia
		Empoasca decipiens (Paoli)	Egypt
		Borogonalia impressifrons (Signoret)	Bolivia
		Macrosteles fascifrons (Stål)	USA
		Nesosteles neglectus (DeLong & Davidson)	USA
		Paratanus exitiosus (Beamer)	Bolivia
		Paratanus yusti Young	Bolivia
	Aphidae	*Hayhurstia atriplicis* (Linnaeus)	USA
		Aphis craccivora Koch	Bolivia, Argentina, Egypt, USA
		Aphis nasturtii Kaltenbach	USA
		Pemphigus (Pemphigus) populi-venais Fitch	USA
		Aphis favae (Scopoli)	Northern Europe
		Aphis gossypii Glover	Egypt
		Macrosiphum euphorbiae (Thomas)	Bolivia, Perú
		Myzus persicae (Sulzer)	Bolivia, Perú
	Triozidae	*Heterotrioza chenopodii* Reuter	Argentina, Peru
	Pentatomidae	*Nezara viridula* Linnaeus	Argentina
		Thyanta (Argosoma) patruelis (Stål)	Argentina
		Thyanta (Phacidium) aeruginosa Berg	Argentina
		Dichelops furcatus (Fabricius)	Argentina
	Lygaeidae	*Nysius natalensis* Evans	South Africa
		Nysius raphanus Howard	USA
		Nysius cymoides (Spinola)	Italy
		Nysius simulans (Stål)	Peru, Argentina
	Coreidae	*Leptoglossus chilensis* (Spinola)	Argentina
	Rhopalidae	*Xenogenus picturatum* Berg	Argentina
		Liorhyssus hyalinus(Fabricius)	Argentina, Peru
	Miridae	*Atomoscelis modestus* (Van Duzee)	USA
		Orthotylus flavosparsus (Sahlberg)	Argentina
		Orthotylus coagulatus (Uhler)	USA
		Lygus hesperus Knight	USA
		Lygus elisus Van Duzee	USA
		Lygus rugulipennis Poppius	Northern Europe
Thysanoptera	Thripidae	*Thrips tabaci* Lindeman	USA
		Frankliniella tuberosi Moulton	USA
		Frankliniella occidentalis (Pergande)	Peru, USA
	Phlaeothripidae	*Haplothrips (Trybomiella) fiebrigi* Priesner	Argentina
Diptera	Agromyzidae	*Liriomyza huidobrensis* (Blanchard)	Peru, Bolivia
		Liriomyza braziliensis (Frost)	Peru
		Amauromyza Karli (Hendel)	USA
	Anthomyiidae	*Pegomya hyoscyami* (Meigen & Panzer)	USA
	Cecydomiidae	*Prodiplosis longifila* Gagné	Peru
Coleoptera	Curculionidae	*Athesapeuta dodonis* Pajni & Kohli	South Africa
		Hypolixus haerens (Boheman)	South Africa
		Gasteroclisus cuneiformis (Fahraeus)	South Africa

		Neocleonus sannio (Herbst, J.F.W.)	South Africa
		Baris amaranthi Marshall	South Africa
		Cosmobaris americana Casey	USA
	Chrysomelidae	*Acalymma demissa* Erichson	Bolivia
		Calligrapha curvilinea Stal	Bolivia
		Cassida nebulosa Linnaeus	Demark
		Diabrotica speciosa (Germar)	Bolivia
		Diabrotica sicuanica Bechyné	Peru
		Chaetocnema concinna (Marsham)	Europe
		Chaetocnema tibialis (Illiger)	Europe
		Phyllotreta pusilla Horn	USA
		Epitrix subcrinita (J. L. LeConte)	Southern Europe
		Epitrix yanazara Bech	Peru, Bolivia
	Meloidae	*Epicauta latitarsis* (Haag-Rutenberg)	Peru
		Epicauta willei Denier	Peru
		Epicauta adspersa Klug	Argentina
		Epicauta atomaria Germar	Argentina
	Melyridae	*Astylus subannulatus* Pic	Peru
	Silphidae	*Aclypea opaca* (Linnaeus)	Europe
Lepidoptera	Gelechiidae	*Eurysacca melanocampta* (Meyrick)	Bolivia, Perú
		Eurysacca quinoae Povolný	Bolivia, Perú
		Eurysacca media Povolný	Argentina
		Scrobipalpa atriplicella (Röslerstamm)	USA, Canada, Demark
	Geometridae	*Perizoma sordescens* Dognin	Bolivia
	Noctuidae	*Agrotis ipsilon* (Hufnagel)	Peru, Bolivia
		Chloridea virescens (Fabricius)	Peru, Bolivia
		Dargida grammivora Walker	Bolivia
		Dargida acanthus (Herrich-Schäffer)	Bolivia
		Feltia experta (Walker)	Bolivia, Peru
		Helicoverpa quinoa Pogue & Harp	Bolivia
		Helicoverpa titicacae Hardwick	Bolivia
		Helicoverpa atacamae Hardwick	Bolivia
		Helicoverpa zea (Boddie)	USA
		Helicoverpa gelotopoeon (Dyar)	Argentina
		Peridroma saucia(Hübner)	Bolivia
		Mythimna unipuncta (Haworth)	Bolivia
		Rachiplusia nu (Guenée)	Argentina
		Trichoplusia ni (Hübner)	USA
		Syngrapha falcifera (Kirby)	USA
		Spodoptera frugiperda (J. E. Smith)	Argentina, Peru, Bolivia
		Spodoptera exigua (Hübner)	USA
		Spodoptera eridania (Stoll)	Bolivia, Peru
		Spodoptera ornithogalli Guenée	USA
		Spodoptera ochrea (Hampson)	Peru
	Crambidae	*Herpetogramma bipunctalis* (Fabricius)	Peru
		Spoladea recurvalis (Fabricius)	Peru
		Achyra bifidalis (Fabricius)	Argentina
	Pyralidae	*Loxostege sticticalis* Linnaeus	USA
		Nomophila indistinctalis (Walker)	Peru
	Erebidae	*Spilosoma virginica* (Fabricius)	Argentina
Trombidiformes	Tetranychidae	*Tetranychus urticae* (Koch)	Argentina

Table S2. Predatory insects that have been reported in quinoa fields in the world.

Order	Family	*Species*	*Type of prey*
Dermaptera	Labiduridae	*Labidura riparia* (Pallas)	Insects that inhabit the soil surfave
Hemiptera	Miridae	*Rhinacloa spp.*	eggs and small larvae of lepidopterans, small arthropods such as thrips, aphids and mites.
	Anthocoridae	*Orius insidiosus* (Say)	
		Orius tristicolor (White)	
		Paratriphleps laeviusculus Champion	
	Berythidae	*Metacanthus tenellus* Stål	
	Geocoridae	*Geocoris* sp.	
	Nabidae	*Nabis punctipennis* Blanchard	
		Nabis argentinus Meyer-Dür	
	Pentatomidae	*Podisus chilensis* (Spinola)	larvae of lepidopterans
		Podisus nigrispinus (Dallas)	
	Reduviidae	*Zelus nugax*	
Neuroptera	Chrysopidae	*Chrysoperla externa* (Hagen)	Aphids and other soft-bodied insects such as thrips, eggs and small larvae of lepidopterans.
		Chrysoperla sp.	
	Hemerobiidae	*Sympherobius* sp.	
		Hemerobius sp.	
Coleoptera	Carabidae	*Calosoma argentinensis* Csiki	Insects that inhabit the soil surfave such as crickets, larvae and pupae of cutworms of the genus Agrotis, Feltia, Peridroma, and those larvae that descend from the plant to the soil to pupate, such as Chrysodeixis includens, Spodoptera spp., Chloridea spp. and Copitarsia spp
		Bemdidium sp.	
		Stenolophus plebejus Dejean	
		Chlaenius sericeus (Forster)	
		Chlaenius sp.	
		Amara sp.	
		Pterostichus sp.	
		Notiobia sp.	
		Blennidus peruvianus (Dejean)	
		Tetracha chilensis (Laporte)	
		Tetragonoderus sp.	
	Coccinellidae	*Cycloneda sanguinea* (L.)	Aphids and thrips
		Hippodamia convergens Guerin	
		Scymnus sp.	
		Eriopis connexa connexa (Germar)	
		Paraneda gutticollis Mulsant	
		Harmonia axyridis (Pallas)	
		Hiperaspis festiva Mulsant	
		Coccinella ancoralis Germar	
		Olla v-nigrun (Mulsant)	
Diptera	Syrphidae	*Allograpta exotica* Wiedemann	Aphids
		Allograpta piurana Shanon	
		Pseudodoros clavatus (Fabricius)	
		Syrphus shorae Fluke	
		Toxomerus sp.	
	Dolichopodidae	*Condylostylus quadricolor* (Walker)	Small insects such as adults of leafmining flies and mites

Table S3. Parasitoids of common pests of quinoa

Order	Family	Species	Host
Diptera	Tachinidae	*Gonia peruviana* Townsend	*Agrotis* spp.
		Gonia pallens Wiedemann	
		Gonia sp.	*Chloridea virescens* (Fabricius)
		Archytas marmoratus (Townsend)	*Agrotis* spp., *Spodoptera* spp., *Chloridea virescens* (Fabricius)
		Linnaemya comta (Fallén)	*Agrotis* spp.
		Prosopochaeta fidelis (Reinhard)	
		Prosopochaeta setosa (Townsend)	*Copitarsia* spp.
		Velardemyia ica Valencia	*Agrotis* spp.
		Winthemia reliqua Cortés & Campos)	*Agrotis* spp., *Spodoptera* spp.
		Eucelatoria australis Townsend	*Spodoptera* spp., *Chloridea virescens* (Fabricius)
		Protogoniops sp.	*Spodoptera* spp.
		Eucelatoria digitata Sabrosky	*Chrysodeixis includens* Walker, *Chloridea virescens* (Fabricius)
		Voria ruralis (Fallén)	*Chrysodeixis includens* Walker
		Lespesia sp.	
		Hemilydella fasciata Townsend	*Chloridea virescens* (Fabricius)
		Euphorocera peruviana Townsed	*Chloridea virescens* (Fabricius), *Copitarsia* spp.
		Patelloa similis (Townsend)	*Copitarsia* spp.
		Incamyia sp.	
		Winthemia sp.	
		Dolichostoma arequipae (Townsend)	
		Peletería robusta (Wiedemann)	
		Phytomyptera sp.	*Eurysacca quinoae* Povolný, *Eurysacca melanocampta* (Meyrick)
Hymenoptera	Ichneumonidae	*Enicospilus* sp.	*Agrotis* spp., *Chloridea virescens* (Fabricius)
		Enicospilus merdarius (Gravenhorst)	*Spodoptera* spp.
		Ophion sp.	*Agrotis* spp.
		Coccygomimus sp.	
		Campoletis flavicincta (Ashmead)	*Spodoptera* spp., *Chloridea virescens* (Fabricius)
		Campoletis curvicauda (Lopez Cristobal)	*Spodoptera* spp.
		Hyposoter sp.	*Copitarsia* spp.
		Netelia sayi (Cushman)	
		Diadegma sp.	*Eurysacca quinoae* Povolný, *Eurysacca melanocampta* (Meyrick)
	Braconidae	*Chelonus* sp.	*Agrotis* spp.
		Meteorus chilensis Porter	
		Cotesia marginiventris (Cresson)	*Spodoptera* spp.
		Chelonus insularis Cresson	
		Rogas sp.	
		Opius sp.	*Liriomyza huidobrensis* (Blanchard)
		Phaedrotoma scabriventris (Nixon)	
		Aphidius matricariae Haliday	*Myzus persicae* (Sulzer), *Macrosiphum euphorbiae* (Thomas)
		Aphidius colemani Viereck	
		Aphidius smithi Sharma & Subba Rao	
		Aphidius rosae Haliday	
		Aphidius sp.	

		Lysiphlebus testaceipes (Cresson)	
		Praon volucre (Haliday)	
		Meteorus sp.	*Eurysacca quinoae* Povolný, *Eurysacca melanocampta* (Meyrick)
		Apanteles sp.	
		Deleboea	
	Scelionidae	*Telenomus remus* (Nixon)	*Spodoptera* spp.
	Encyrtidae	*Copidosoma truncatellum* (Dalman)	*Chrysodeixis includens* Walker
		Copidosoma gelechiae Howard	*Eurysacca quinoae* Povolný, *Eurysacca melanocampta* (Meyrick)
	Platygasteridae	*Synopeas sp.*	*Prodiplosis longifila* Gagné
	Eulophidae	*Euplectrus platyhypenae* Howard	*Chrysodeixis includens* Walker, *Copitarsia* spp.
		Closterocerus cinctipennis Ashmead	*Liriomyza huidobrensis* (Blanchard)
		Chrysocharis flacilla (Walker)	
		Chrysocharis sp.	
		Derostenus sp.	
		Diglyphus websteri (Crawford)	
		Diglyphus *begini* (Ashmead)	
		Zagrammosoma multilineatum (Ashmead)	
		Agromyzophagus sp.	
	Pteromalidae	*Halticoptera arduine* (Walker)	
		Halticoptera sp.	

Table S4. Bioinsecticides used on quinoa pests

**Bioinsecticide**	**Quinoa Pest controlled**
Bacillus thuringiensis var *kurstaki*	*Spodoptera eridania*<br>*Spodoptera frugiperda*<br>*Chloridea virescens*<br>*Eurysacca melanocampta*<br>*Chrysodeixis includens*<br>*Spoladea recurvalis*<br>*Thrips tabaci*
Bacillus thuringiensis var *aizawai*	*Spodoptera frugiperda*<br>*Copitarsia corruda*
Spinosad	*Spodoptera frugiperda*<br>*Spodoptera eridania*<br>*Copitarsia corruda*<br>*Chloridea virescens*<br>*Chrysodeixis includens*<br>*Frankliniella occidentalis*
Tetraethyl silicate	*Frankliniella occidentalis*
